# Dengue hemorrhagic fever presenting as encephalitis: a case report

**DOI:** 10.1186/s13256-019-2201-x

**Published:** 2019-09-06

**Authors:** W S Weerasinghe, Arjuna Medagama

**Affiliations:** 1 Teaching Hospital Peradeniya, Peradeniya, Sri Lanka; 2 Department of Medicine, Faculty of Medicine, University of Peradeniya, Peradeniya, Sri Lanka

**Keywords:** Dengue hemorrhagic fever, Dengue encephalitis, Seizure, Hemiparesis

## Abstract

**Background:**

Dengue fever and dengue hemorrhagic fever incidence is increasing in Sri Lanka, especially among the young population. Uncommon presentations of this common illness make diagnostic dilemmas and can delay standard treatment which leads to unfavorable outcomes.

**Case presentation:**

An 18-year-old Sri Lankan Sinhalese boy presented with a history of 1 day of fever and an episode of seizure followed by left-side hemiparesis. He was diagnosed to have dengue complicated by dengue hemorrhagic fever and recovered with minimal residual weakness. He presented with neurological symptoms; cerebrospinal fluid analysis, electroencephalogram, and magnetic resonance imaging showed evidence of encephalitis. Positive dengue antigen and antibody in serum and cerebrospinal fluid with the exclusion of other possible etiologies confirmed parainfectious dengue encephalitis. He was started on sodium valproate 200 mg 8 hourly and made a slow neurological recovery with mild residual weakness (grade 4+ power) in his left upper limb at 2 months with intensive supervised physiotherapy.

**Conclusion:**

Standard national guideline-based management of dengue illness has significantly reduced its mortality rates in Sri Lanka. However, uncommon presentations of a common illness often cause diagnostic dilemmas. Hence, reporting of these presentations and knowing the epidemiologic patterns of the disease help physicians to arrive at the correct diagnosis even though they do not have sophisticated serological investigations. Overall, this can improve the quality of health care and reduce mortalities, especially in a resource-poor setup.

## Background

Dengue fever (DF) is a leading cause of morbidity in Sri Lanka and 185,688 suspected cases of dengue were reported to the Epidemiology Unit, Ministry of Health from all over the island during 2017 [1]. Approximately 41% of dengue cases were reported from the Western Province of Sri Lanka.

The first serologically confirmed case of dengue was reported in 1962 and the first documented dengue outbreak occurred in 1965–1966 [2]. DF and dengue hemorrhagic fever (DHF) comprise the bulk of symptomatic illness while dengue encephalitis is a rare entity (around 4 to 21%) [3].

## Case presentation

We report the case of a previously well 18-year-old Sri Lankan Sinhalese boy, resident of a dengue endemic area, who presented with a 1-day history of high fever and tonic-clonic movements of the left upper and lower limbs later converting into a generalized tonic-clonic (GTC) seizure to the Teaching Hospital Peradeniya, Sri Lanka. The fever was high grade without chills but associated with arthralgia, myalgia, headache, and vomiting. The seizures commenced on the evening of the first day of the illness, lasted for 10 minutes and were associated with postictal drowsiness. A persistent left-sided face, arm, and leg weakness was apparent as the postictal drowsiness improved. There were no associated sensory symptoms and the weakness was more pronounced in his face and upper limb. There was no associated abdominal pain, postural dizziness, reduced urine output, or any bleeding tendency. There was no recent history of vaccination and no skin rashes. He had been investigated following a head injury 10 months back. He presented after a road traffic accident with mild drowsiness without any focal neurological weakness and a non-contrast computed tomography (NCCT) scan of his brain had been normal. He was completely well on discharge and no long-term neurological symptoms were evident until this incident. His past medical history was unremarkable with no history of epilepsy or collagen vascular diseases.

A general examination revealed a temperature of 38.33 ºC (101 ºF) but was otherwise unremarkable. A neurologic examination revealed our patient to be drowsy but arousable, without signs of meningism. A conscious level corresponding to Glasgow Coma Scale (GCS) of 10/15 (E-4, V-1, M-5) was present with horizontal gaze palsy to the left, and normally reactive pupils of 3 mm. A cranial nerve examination revealed facial nerve palsy of upper motor neuron type on the left with flaccid paralysis of his left upper limb (power 0/5) and diminished left lower limb (power 2/5) power. Deep tendon reflexes were diminished on the left with hypotonia. Plantar response was extensor on the left side. No cerebellar signs were apparent.

His vital signs were stable with a pulse rate of 100 beats per minute (bpm) and blood pressure of 107/70 mmHg. No right hypochondriac tenderness or murmurs were present, and the rest of the examination was unremarkable.

Initial investigations are summarized in Table 1. An urgent NCCT of his brain revealed no evidence of infarction or intracerebral hemorrhage (ICH). An interval NCCT and contrast-enhanced computed tomography (CECT) scan was also performed and did not show any infarction, cerebral abscess, or space-occupying lesion. We performed a lumbar puncture (LP) and cerebrospinal fluid (CSF) was colorless and clear: total white blood cell (WBC), 03 cells/mm^3^ (lymphocytes); red blood cell (RBC), 00 cells/mm^3^; CSF protein, 250 mg/L; CSF sugar, 3.4 mmol/l; random blood sugar (RBS), 5.7 mmol/l; CSF Gram stain and bacterial cultures were negative. CSF viral studies were not performed due to the small volume of CSF being available at the first LP and a repeat attempt was not made in the context of dropping platelet (PLT) counts.

**Table 1:** Initial investigations

Investigation	Results
White blood cells (WBC)	12,070/mm^3^ (N, 90.3%; L, 7.5%)
Hemoglobin (Hb)	13.5 g/dl
Pack cell volume (PCV)	40.6%
Platelets (PLT)	189,000/mm^3^
Aspartate aminotransferase (AST)	31.5 U/L
Alanine aminotransferase (ALT)	30.8 U/L
Serum creatine (S. Cr)	128 umol/L
Serum albumin (Alb)	44 g/dL
Serum sodium (Na)	136 mmol/L
Serum potassium (K)	3.8 mmol/L
Magnesium (Mg)	1.07 mmol/
Corrected calcium (Ca)	2.4 mmol/L
Random blood sugar (RBS)	5.7 mmol/L
C-reactive protein (CRP)	3.9 mg/L

Electrocardiography, urine full report, clotting profile, and chest X-ray were normal

*L* lymphocytes, *N* neutrophils

**Fig. 1:**
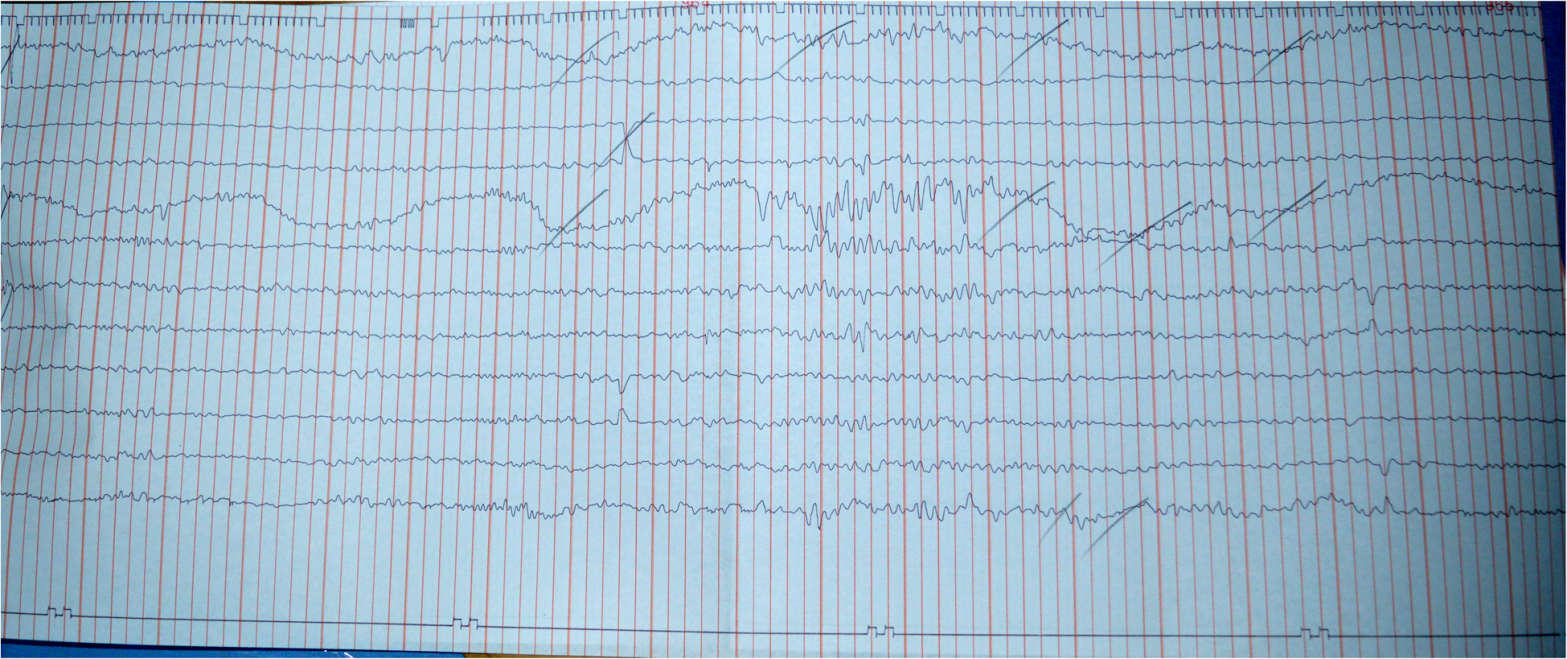
An electroencephalograph showing generalized slow waves with burst of activity in right frontotemporal region

Electroencephalography (EEG) performed on the following day showed generalized slow waves with a burst of activity in the right frontotemporal region compatible with organic brain disease (Fig. 1).

**Fig. 2:**
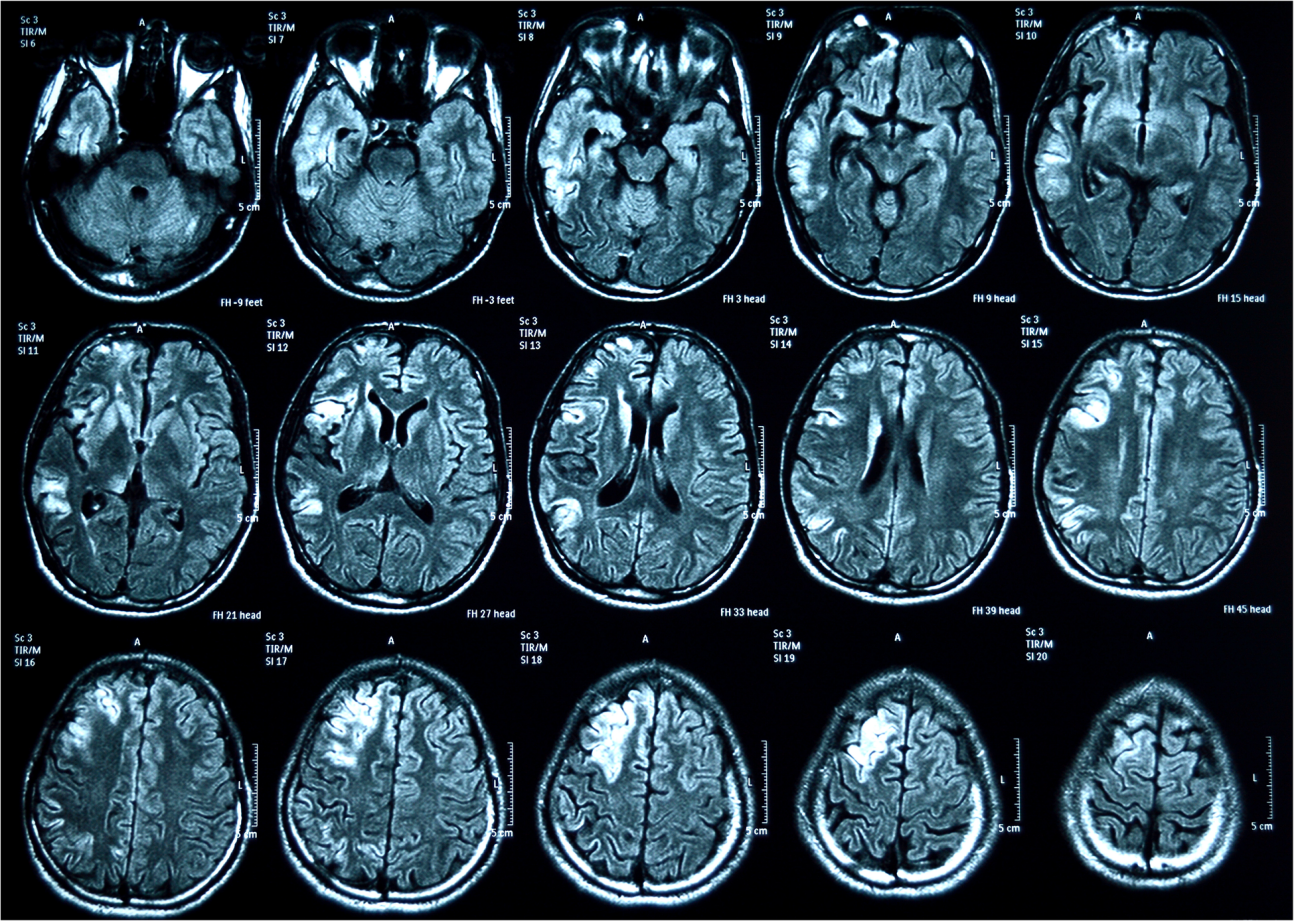
Magnetic resonance (fluid-attenuated inversion recovery) images with some faint meningeal enhancement appreciated in right frontotemporal area

A magnetic resonance imaging (MRI) of his brain was performed which showed abnormal high intensity subcortical white matter and cortical gray matter in right frontoparietal and temporal lobes in T2-weighted (T2W) and fluid-attenuated inversion recovery (FLAIR) images with some faint meningeal enhancement appreciated in right frontotemporal area suggestive of right-sided meningoencephalitis (Fig. 2).

A presumptive diagnosis of viral encephalitis was made, and he was started on intravenously administered acyclovir 500 mg 8 hourly and intravenously administered ceftriaxone 2 g 12 hourly with intravenously administered dexamethasone 4 mg 8 hourly and sodium valproate 200 mg 8 hourly. Supportive care with nasogastric feeding, urine catheterization, and intravenously administered fluids was also started, and he was continuously monitored within the high dependency unit to identify clinical or biochemical deterioration.

On the fifth day of illness, fever was still present, neurological signs remained unchanged, and rising liver transaminases were noted, that is, aspartate aminotransferase (AST) of 4918 U/L and alanine aminotransferase (ALT) of 2987 U/L, together with leukopenia and thrombocytopenia (WBC, 3770 cells/μl; PLT, 23,000 cells/μl). A peripheral blood film was found to be compatible with a viral infection without features of microangiopathic hemolytic anemia (MAHA).

The marked rise in transaminases together with leukopenia and thrombocytopenia prompted a fresh search for an alternative diagnosis and serum dengue nonstructural protein 1 (NS1) [4] antigen was performed which was positive. Testing CSF for dengue Immunoglobulin M (IgM) with enzyme-linked immunosorbent assay (ELISA) antibody and NS1 antigen was not possible at this moment as the initial CSF sample was inadequate. The viral studies performed considering the possible neurotrophic viruses in the serum on the seventh day of the illness and varicella-specific IgM, cytomegalovirus (CMV) IgM, and Epstein–Barr virus (EBV) IgM (ELISA method) were negative. Serum antibody testing for enterovirus and coxsackievirus was not feasible in the government sector and our patient could not afford to take the test from the private sector.

National guidelines [5]-directed dengue monitoring and management were commenced. On day 5 of the illness a rising pack cell volume (PCV), with ultrasonographic evidence of free fluid in the hepatorenal pouch and gallbladder wall edema corresponding to plasma leakage of dengue critical phase, was found. Table 2 demonstrates the laboratory results during the hours spent in the critical phase. He made a full recovery from dengue critical phase 48 hours after confirming DHF. Serum dengue IgM was positive on day 7 of the illness but IgG was negative.

**Table 2:** The laboratory results during the hours spent in the critical phase

	WBC (10^3^ cells/μl)	NEU (%)	LYM (%)	PCV (%)	Hb (g/dl)	PLT (10^3^cells/μl)	AST (U/L)	ALT (U/L)	Ca (mmol/L)
00 hour	4.30	76.6	14.2	48.4	15.7	42	2545	1097	2.4
12 hours	7.5	63.7	24.5	49	16.2	23	1836	672	
24 hours	7.92	62.8	27.3	47.4	16.3	21	1679	566	
36 hours	8.38	59.7	31.1	44	15.1	45	1400	338	2.18
48 hours	9.21	66.4	26.2	41	14.2	49	1166	274	
72 hours	12.12	80.9	12.8	40.1	13.9	101	780	118	

*ALT* alanine aminotransferase, *AST* aspartate aminotransferase, *Ca* corrected calcium, *Hb* hemoglobin, *LYM* lymphocytes, *NEU* neutrophils, *PCV* pack cell volume, *PLT* platelets, *WBC* white blood cells

He made a slow recovery with mild residual weakness (grade 4+ power) in his left upper limb at 2 months with intensive supervised physiotherapy.

**Fig. 3:**
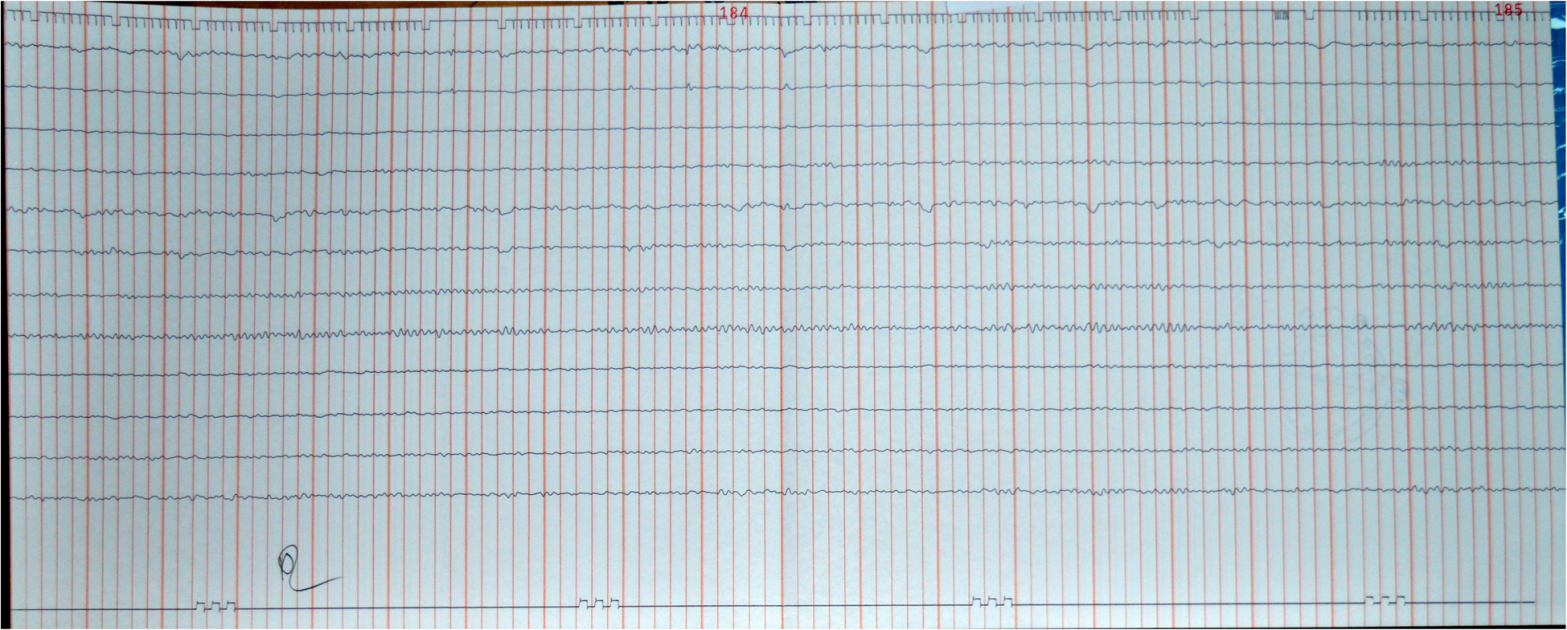
Normal electroencephalograph

Considering his slow recovery, a CSF analysis was repeated at 2 months and showed total WBC, 04 cells/mm^3^ (lymphocytes); RBC, 00 cells/mm^3^; CSF protein, 540 mg/L; CSF sugar, 3.4 mmol/L; RBS, 5.7 mmol/L; adenosine deaminase (ADA), 3.0 U/L; CSF Gram stain and bacterial cultures were negative. Dengue IgG (ELISA) was positive in CSF and IgM (ELISA) was negative. Since full virologic profile was not performed in the first presentation, CSF was also tested for other neurotrophic viruses such as herpes simplex virus (HSV) by polymerase chain reaction (PCR), HSV-1 and HSV-2 antibodies, Japanese encephalitis (JE) antibody, enterovirus, and coxsackievirus. All the CSF studies and serum for human immunodeficiency virus (HIV) screening were negative. A repeat EEG was also performed, and it was normal (Fig. 3).

He had been followed up at our medical clinic since discharge, where a gradual improvement in his weakness was evident. After 12 months of follow-up, he showed remarkable recovery of his neurologic functions without any residual weakness.

## Discussion and conclusion

Dengue is the commonest arthropod-borne viral infection in humans [6]. It is caused by a family of positive, single-stranded, enveloped ribonucleic acid (RNA) viruses called *Flaviviridae*, genus *Flavivirus* [7]. There are four closely related but antigenically different serotypes of the virus that can cause dengue (DEN-1, DEN-2, DEN-3, DEN-4). Dengue has a wide spectrum of infection outcomes (asymptomatic to symptomatic). Symptomatic illness can vary from undifferentiated fever (viral syndrome), DF, DHF, and dengue with unusual manifestations such as isolated organopathies [5, 8].

Expanded dengue syndrome/isolated organopathy is an entity in which some patients present with unusual manifestations of organ involvement with or without fluid leakage [5]. Encephalitis and meningoencephalitis have been found in 4–21% of cases of dengue [3]. This wide variation in the prevalence of encephalitis most likely reflects differences in the populations studied and the use of different criteria to define dengue encephalitis. Distinguishing dengue encephalitis from other types of central nervous system (CNS) involvement is also challenging [3].

Dengue is classically thought to be a non-neurotropic virus but DEN-2 and DEN-3 are the most frequently involved serotypes in neurotropism [9–11]. Dengue encephalopathy is a secondary manifestation of the DF frequently associated with multisystem derangement found later in the course of the illness, which is thought to be due to an immune-mediated inflammatory response of the body [12]. Conversely, encephalitis occurs due to direct invasion of the virus and occurs in the viremic phase of the illness as in this patient. Although it is a rare occurrence, autopsy studies have proved the presence of virus antigen in brain parenchymal cells using immunoperoxidase stain [13].

There were many reported cases of dengue encephalitis and other neurological manifestations by Kularatne *et al.* from Sri Lanka [14], by Misra *et al*. from India [15], and Solomon *et al*. from Vietnam [16]. All of them had neurological symptoms with signs such as headache, confusion, seizures, hemiparesis, and even coma. Some of them described EEG changes and MRI changes depending on the area of the brain involved.

Bhoi *et al.* [17] studied a cohort of 21 patients with dengue encephalitis in a study conducted to evaluate MRI and computed tomography (CT) changes and showed hyperintensities in MRI of brains involving bilateral parietal region, corona radiata, internal capsule, centrum semiovale bilaterally, basal ganglia, and thalamus. Carod-Artal *et al.* [18] summarized findings of eight autopsy studies reported on fatal cases of dengue with neurological involvement which showed histopathologic patterns of cerebral edema, congestion, hemorrhage, perivascular lymphocytes infiltration, and even necrosis of the brain matter. There are many case reports and studies found in the literature proving that various parts of the brain can be affected in different ways [19–21]. MRI of our patient’s brain showed hyperintensities in subcortical white matter and cortical gray matter in right frontoparietal and temporal lobes, and some faint meningeal enhancement in the right frontotemporal area. These lesions are compatible with the clinical signs shown. The interesting fact is that none of them follow a uniform pattern which helps a clinician to suspect dengue by looking at MRI or CT.

Carod-Artal *et al.* [18] proposed three criteria to define dengue encephalitis. They are: the clinical signs and symptoms of CNS involvement; demonstration of dengue virus RNA, IgM, or NS1 antigen in CSF; and CSF pleocytosis without other neuroinvasive pathogens. The literature also showed that the presence of dengue antibody in CSF can be considered a piece of firm evidence of CNS invasion, but the practical difficulty in performing a LP in a patient with dengue was also highlighted [22]. Most of the cases reported in the literature [23] were based on clinical diagnosis of encephalitis followed by serological confirmation of dengue infection and exclusion of the other neurotropic viruses such as HSV, CMV, EBV, JE, enterovirus, and coxsackievirus [24]. MRI or CT is nonspecific but often provided valuable clues for further investigations and CSF studies. This fact further highlights the need for more evidence and studies to access the sensitivity and specificity of these investigations to diagnose dengue encephalitis.

A wide variety of symptoms are documented in the literature as the initial presentation of dengue. Sometimes it is very difficult to suspect dengue encephalitis by history, examination, and initial investigations, as in this patient. This kind of atypical presentation of DF can lead to diagnostic delays and adverse patient outcomes.

This case report emphasizes the importance of considering dengue as a differential diagnosis in a patient with features of encephalitis especially in a background of dengue epidemic and endemicity, as it changes the monitoring and management. Dengue NS1 antigen is an affordable investigation in developing countries like Sri Lanka and is sometimes available in the government sector. A combination of clinical signs and other basic investigations like WBC, PLT, PCV, and liver enzymes will provide clues to diagnosis and the proper monitoring of the patient to prevent the life-threatening complication of dengue illness in an atypical presentation like this. Another important fact is that although we have national guidelines to manage DF, we must individualize the plan of management for this kind of patient.

## Data Availability

Not applicable.

## Abbreviations

ADA: Adenosine deaminase
ALT: Alanine aminotransferase
AST: Aspartate aminotransferase
bpm: Beats per minute
CECT: Contrast-enhanced computed tomography
CMV: Cytomegalovirus
CNS: Central nervous system
CSF: Cerebrospinal fluid
CT: Computed tomography
DF: Dengue fever
DHF: Dengue hemorrhagic fever
EBV: Epstein–Barr virus
EEG: Electroencephalography
ELISA: Enzyme-linked immunosorbent assay
FLAIR: Fluid-attenuated inversion recovery
GCS: Glasgow Coma Scale
GTC: Generalized tonic-clonic
HIV: Human immunodeficiency virus
HSV: Herpes simplex virus
ICH: Intracerebral hemorrhage
JE: Japanese encephalitis
LP: Lumbar puncture
MAHA: Microangiopathic hemolytic anemia
MRI: Magnetic resonance imaging
NCCT: Non-contrast computed tomography
NS1: Nonstructural protein 1
PCR: Polymerase chain reaction
PCV: Pack cell volume
PLT: Platelet
RBC: Red blood cell
RBS: Random blood sugar
T2W: T2-weighted
WBC: White blood cell
